# Injurious and Adulterated Beverages

**Published:** 1887-04

**Authors:** 

**Affiliations:** Montreal


### INJURIOUS AND ADULTERATED BEVERAGES.

BY AN OCCASIONAL CONTRIBUTOR, MONTREAL.

Considerable attention has been directed of late years to the innumerable ways in which food and beverages are being adulterated, and to the deceptions practiced by merchants and retailers to palm off upon the public spurious imitations in lieu of the genuine article. To attempt to mention in detail, or fully describe the "tricks of trade" in this respect, would require a volume of ponderous dimensions. It is proposed, however, to notice briefly the ingredients and properties of some of our most generally used beverages, tea, coffee, etc., with a description of a few of the adulterants used in manufacturing their numerous imitations.

Indications are not wanting, throughout our large cities, that the public are becoming slowly aroused to the dangers attending the use of these trashy and pernicious imitations, also to the debilitating and demoralizing effects which invariably attend the frequent use of them. A little judicious testing and experiment will suffice to show how very rarely the genuine unadulterated article is procurable.

The laws in some countries, which prohibit the adulteration of food, even as they now stand, would be a tolerably good protection, if they were strictly administered, but in general, they are but very little more than a dead letter. Moreover, it seems to be "nobody's business" specially to insist upon their enforcement.

In the few cases which have arisen among retailers, so many formalities are required, so much time has been lost, and the prosecution has proved such a troublesome and disagreeable business, that the experiment is seldom repeated.

It was only recently that a score of small tin boxes, closely resembling those used for packing gunpowder, stood on a table in the Appraiser's office, at No. 402 Washington street. Near them sat Mr. James R. Davies, inspector of tea for the port of New York.

"So you cannot guess what they are," said the Inspector to a bystander. "Well, they are samples of different kinds of tea sent to me for inspection. It is no easy job, I can assure you, as you will understand when I tell you that within the past thirty days we have rejected at this port 2,000 packages of tea from China and Japan, 200 from Canada and about 4,000 from London. No article can be adulterated so easily as tea. The genuine stuff can be mixed either with leaves that are spurious or exhausted by age and fermentation, or with such chemicals and other deleterious substances as render it entirely unfit for use.

"The rejected tea is sent back to the ports from which it came. This has been the rule since March 2, 1883, when the act to prevent the importation of adulterated and spurious teas came into force."

"The greater my experience becomes," writes Dr. Clonston, in the Annual Report of the Royal Edinburgh Asylum for the Insane, "the more I urge the substitution of milk for stimulants. In very acute cases, both of depressions and exaltations, where the disordered working of the brain tends rapidly to exhaust the strength, I rely more and more on milk and eggs made into liquid custards. One such case this year got 8 pints of milk and 16 eggs daily for three months, and recovered under this treatment.

"The patient was almost dead on admission, acutely delirious; absolutely sleepless, and very nearly pulseless." The cup of tea so much in demand by many women when tired, should be exchanged for *milk, eggs and rest.*

Dr. Felix L. Oswald, of New York, in his "Physiological effects of the Poison Problem," says on this subject: "But there are other forms of physical degeneration which can with certainty be ascribed to the influence of the secondary stimulant. Tobacco makes the Turks indolent, tea and coffee make us nervous and dyspeptic, and the worst of it is that these minor vices pave the way to ruin; a constitution enfeebled by theine poison, is less able to resist the influence of fusel poison. It is a great mistake to suppose that abstinence from concentrated alcoholic liquors could atone for the habitual use of other stimulants."

To the *astringent principle* or *tannine,* which forms from thirteen to eighteen per cent. of the dried leaf, tea owes its astringent taste, its constipating effect upon the bowels, and its property of communicating an ink-like color to water containing salts of iron. The tannin also produces an exhilarating effect, and is the principal ingredient of the Indian betel-nut, which produces a mild and agreeable form of intoxication.

As on most other subjects, physicians disagree on some points in regard to the effects of tea, but all agree, that as a general rule, it is very bad for minors whose growth is not completed. When used medicinely in fevers and some forms of heart disease, it is often of temporary service, and a very strong decoction has often proved servicable as an antidote in some cases of poisoning, and in allaying the drunkards's craving for liquor. It is impossible to speak too strongly against the habit occasionally adopted by students of keeping off their natural sleep by the frequent use of strong tea. The persistent indulgence in such a habit is certain to lead to the utter destruction of both bodily and mental vigor.

Apropos of the depressing after-effects of tea, upon a nervous constitution and temperament, there has recently occurred a case in print, where a patient from Canada, suffering from nervous depression, called upon a noted Elaym physician, to consult with him in regard to his ailments.

He naturally expected a formidable prescription and a long course of drugs and medicines, instead of which, he was simply recommended to abandon *tea;* and after having done so, in obedience to the advice given, he soon recovered his former health and spirits.

If the foregoing should be disputed, let them be put to test by inquiring in proper quarters and by consulting the family physician ; the result will most probably be its entire confirmation in essential particulars, unless the medico should be one of those described by the poet as—"suiting his physic to his patient's taste," in which case he would perhaps modify his opinion, not wishing to run in opposition altogether to preconceived prejudices. Tastes too long gratified and habits formed at too early an age, and continued throughout youth and maturity, cannot be lightly changed or abandoned ; but if one cannot or will not give up tea, at all events, spare the children such inhuman wrong and injury as result from its early and frequent use.

To the honor of the medical profession, be it recorded that they have spoken with no uncertain voice on the subject of tea and its injurious effects, but in most cases their advice has either been unheeded or forgotten, perhaps only partially believed in, and the results braved for the sake of a passing sensual enjoyment or stimulating habit, too long practiced to be given up without a struggle involving the exercise of firmness and self-denial.

